# A personalized prognostic model based on preoperative body composition and nutritional parameters for gastric cancer patients receiving neoadjuvant chemotherapy

**DOI:** 10.3389/fimmu.2026.1759292

**Published:** 2026-03-13

**Authors:** Zongsheng Sun, Zhengzhao Wang, Ruiqing Liu, Mingyu Yang, Hanhui Jing, Xuesen Li, Shunli Liu, Yuandi Wang, Shanglong Liu, Dongsheng Wang

**Affiliations:** 1 Department of Gastrointestinal Surgery, Affiliated Hospital of Qingdao University, Qingdao, China; 2 Department of Radiology, Affiliated Hospital of Qingdao University, Qingdao, China

**Keywords:** body composition, gastric cancer, model, neoadjuvant chemotherapy, nutrition, prognostic

## Abstract

The long-term survival of patients with locally advanced gastric cancer (LAGC) undergoing neoadjuvant chemotherapy (NAC) remains suboptimal. In this retrospective study, we analyzed 403 NAC-LAGC patients followed by radical gastrectomy between January 2016 and December 2023. The cohort was randomly divided into a training set and a validation set in a 7:3 ratio. Variables with a univariable P value below 0.20 were first identified, and LASSO regression together with stepwise Cox proportional hazards regression was then applied to screen the candidate predictors (p < 0.10). This process yielded the final set of predictive factors based on multidimensional indicators related to nutritional status and body composition. Using the training set, we constructed separate nomograms for predicting overall survival, progression-free survival, and disease-free survival, and then developed a corresponding risk stratification model. Model performance was assessed with Kaplan-Meier survival analyses and the area under the receiver operating characteristic curve, and was further examined in the validation set. Through feature selection, we identified several independent prognostic predictors. Kaplan-Meier survival analyses confirmed that each variable was significantly associated with poor prognosis (p < 0.01). Based on these predictors, we first constructed individual nomograms to predict OS, PFS, and DFS, all of which achieved favorable discriminative performance with AUC values exceeding 0.800. To further enhance risk stratification, we subsequently developed a comprehensive prognostic risk stratification model (PRSM). The PRSM demonstrated robust and reliable predictive ability: in the training cohort, all AUCs were above 0.800 (p < 0.001), with a c-index of 0.836; in the validation cohort, AUCs similarly exceeded 0.800 (p < 0.001), with a c-index of 0.829. Decision curve analysis further indicated that, within an appropriate threshold range, the PRSM provided meaningful clinical net benefit for NAC-LAGC patients. In conclusion, we developed and validated PRSM that incorporates multidimensional predictors reflecting nutritional status and body composition to estimate long-term outcomes in NAC-LAGC patients. The model provides reliable risk stratification and may serve as a practical tool to support individualized nutritional optimization and postoperative management in clinical practice.

## 1 Introduction

Although the global incidence and mortality of gastric cancer have decreased over recent decades, the disease still ranks fifth in both incidence and mortality and remains the second leading cause of cancer-related deaths worldwide (1, 2). Most patients present with locally advanced gastric cancer (LAGC), for which radical surgical resection continues to be the cornerstone of curative therapy. According to the National Comprehensive Cancer Network (NCCN) guidelines, neoadjuvant chemotherapy (NAC) has become a standard treatment option for LAGC, as it can reduce tumor burden, facilitate downstaging, increase the likelihood of achieving an R0 resection, and ultimately improve overall survival (OS) (3, 4). Previous studies have demonstrated that in NAC-LAGC patients, the R0 resection rate can reach approximately 82%, with 3-year and 5-year OS rates of 59% and 53%, respectively (5).

Despite its clinical effectiveness, NAC has not fully translated into satisfactory long-term postoperative survival outcomes in LAGC patients. This limitation is thought to be associated with multiple adverse factors, particularly the negative impact of NAC on patients’ nutritional status. Gastrointestinal toxicities can impede adequate nutrient intake, while the catabolic stress induced by chemotherapy may contribute to metabolic derangements such as hypoalbuminemia and sarcopenia (6). Accordingly, comprehensive preoperative nutritional assessment is essential for prognostic risk stratification and for guiding perioperative nutritional interventions. Among available tools, systematic nutritional indices such as the nutritional risk index (NRI) and prognostic nutritional index (PNI) have demonstrated significant clinical utility in predicting outcomes across various malignancies (7, 8). Przekop et al. reported that patients with low NRI scores exhibited a higher risk of mortality, and that NRI outperformed traditional parameters such as BMI as a prognostic indicator (9). In addition, Ding et al. showed that the PNI score is valuable in predicting both treatment response and survival outcomes in patients with LAGC receiving XELOX-based neoadjuvant therapy combined with anti-PD-1 immunotherapy (7).

Body composition assessment based on computed tomography (CT) has emerged as a widely used method for preoperative nutritional evaluation, as it enables accurate quantification of nutrition-related information reflected by deep anatomic structures (10). Lin et al. demonstrated that both a low skeletal muscle index (SMI) and perioperative SMI loss were independent predictors of suboptimal tumor regression in NAC-LAGC patients (11). CT-derived indices such as the visceral fat index (VFI), intramuscular fat index (IFI), subcutaneous fat index (SFI), and SMI provide objective measurements of muscle and fat distribution as well as their dynamic changes during the perioperative period. These indicators are considered direct surrogates of nutritional status and have been validated as prognostic markers in multiple malignancies, including colorectal and bladder cancer (12, 13). Compared with conventional nutritional indicators, such as the Nutritional Risk Screening 2002 (NRS-2002) and serum albumin, CT-based body composition parameters offer superior sensitivity and are not confounded by inflammation, infection, or fluid retention. They also enable early identification of “latent sarcopenia” and other occult metabolic disorders. A growing body of evidence indicates that these metrics are independent predictors of postoperative complications, recurrence risk, and long-term survival (14–16). Therefore, body composition analysis plays a critical role in prognostic evaluation and perioperative management, providing a scientific basis for individualized nutritional interventions in clinical practice.

To date, there is no risk stratification tool that integrates preoperative nutritional assessments with body composition to predict prognosis in NAC-LAGC patients. This study aims to establish a practical model combining these parameters to predict long-term survival and guide individualized perioperative nutritional interventions.

## 2 Materials and methods

### 2.1 Patients and research design

**Figure 1:**
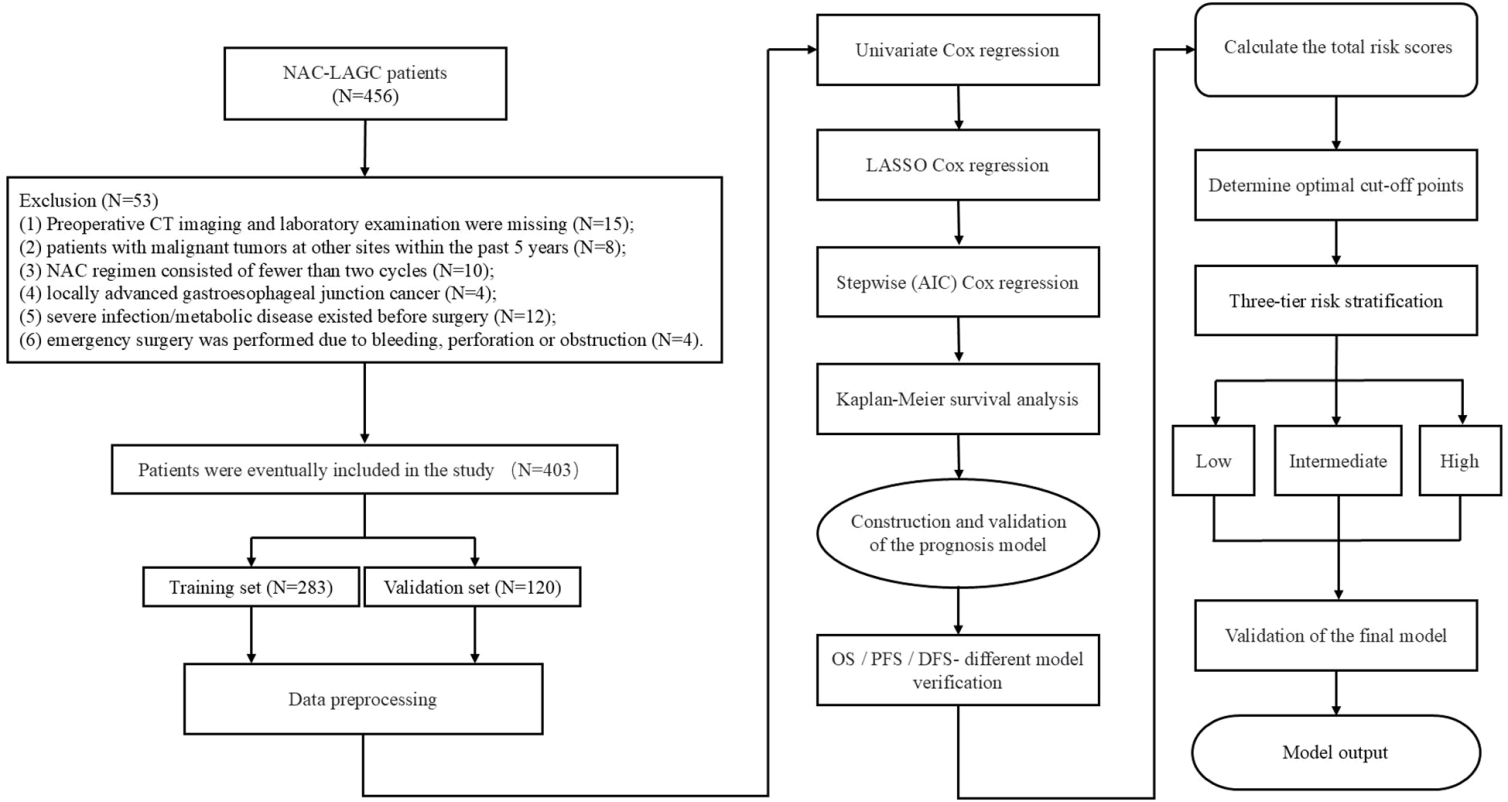
Flowchart of patient selection and analytical process.

Patients diagnosed with LAGC who received NAC at Qingdao University Affiliated Hospital between January 2016 and December 2023 were retrospectively reviewed. The exclusion criteria were as follows: (1) absence of preoperative CT imaging or laboratory tests; (2) history of other malignant tumors within 5 years; (3) completion of fewer than two NAC cycles; (4) diagnosis of advanced esophagogastric junction cancer; (5) presence of severe infection or metabolic disorders before surgery; and (6) requirement for emergency surgical intervention due to bleeding, perforation, or obstruction. The predominant NAC regimens were FLOT (fluorouracil, leucovorin, oxaliplatin, and docetaxel) and SOX (S-1 plus oxaliplatin). In accordance with the 5th edition of the Japanese Gastric Cancer Treatment Guidelines, patients underwent radical gastrectomy with D2 lymphadenectomy approximately 4 weeks after completion of NAC (17). All procedures were performed by experienced gastrointestinal surgeons following standardized protocols. A total of 403 eligible patients were ultimately enrolled after applying the inclusion and exclusion criteria. All patients were prospectively followed, and the primary endpoints included 2-, 3-, and 5-year overall survival (OS), progression-free survival (PFS), and disease-free survival (DFS). The cohort was randomly divided into a training set (n = 282) and a validation set (n = 121) at a ratio of 7:3 for model development and validation. This study adhered to the principles of the Declaration of Helsinki and received approval from the Ethics Committee of Qingdao University Affiliated Hospital (approval number: QYFY WZLL 30251). A schematic overview of the study design is presented in **Figure 1**.

### 2.2 Data collection and prognosis follow-up

This study collected comprehensive baseline clinical data, including gender, age, yp TNM stage, NRS - 2002 score, BMI before and after NAC, ASA score, comorbidities, tumor size, surgical method, intraoperative blood loss, etc. The postoperative specimens were evaluated by professional pathologists, and the tumor size, margin status, pathological stage and tumor regression grade (Tumor Regression Grade, TRG) were recorded (18). Laboratory tests were conducted within one week before the surgery, including CEA, CA19-9, CA12-5, neutrophil count, lymphocyte count, hemoglobin, albumin, platelet count, and globulin. The CT imaging data of the upper abdomen were selected from the relevant images obtained during the re-examination (for NAC efficacy assessment) after the last cycle of NAC was completed, and were saved as DICOM. The calculation methods for inflammation-nutrition related indicators are as follows: neutrophil-lymphocyte ratio (NLR) = N/L, platelet-lymphocyte ratio (PLR) = P/L, systemic immune-inflammation index (SII) = P × N/L, PNI = Albumin (g/L) + 5 × L (10^9^/L), NRI = 1.519 × Albumin (g/L) + 41.7 × (Post-NAC Body Weight/Pre-NAC Body Weight), N = neutrophil, L = lymphocyte, P = platelet. The controlling nutritional status (CONUT) score is based on three routine laboratory indicators: serum albumin, total lymphocyte count, and total cholesterol. Each indicator is scored according to the measured value (0–6 points), and the sum of the three scores is the total score (19). Follow-up was conducted through outpatient visits or telephone interviews according to a standardized schedule: every 3 months during the first 2 years after surgery, every 6 months between years 2 and 5, and annually thereafter, with follow-up censored in June 2025. The survival endpoints were defined as follows. Overall survival (OS) was measured from the date of surgery to death from any cause. Disease-free survival (DFS) was defined as the time from surgery to the first occurrence of tumor recurrence, metastasis, or death. Progression-free survival (PFS) referred to the interval from surgery to the earliest radiologically confirmed disease progression.

### 2.3 Body composition assessment based on CT

**Figure 2:**
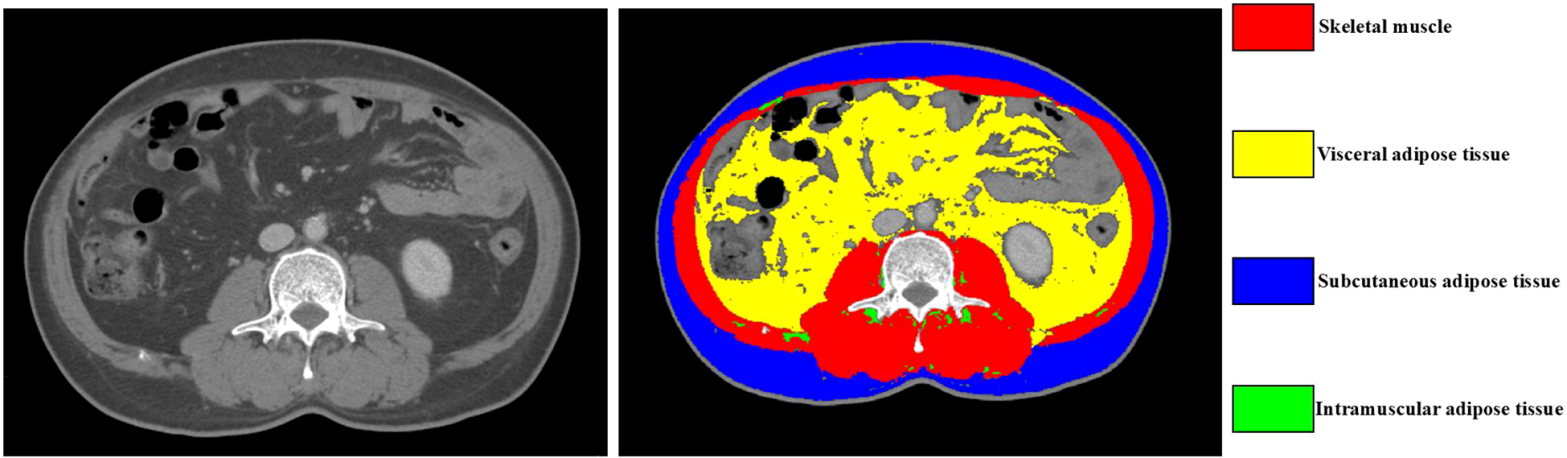
Body composition schematic diagram. The cross-sectional CT image of the third lumbar vertebra is used to quantify body composition variables. The red, yellow, blue, and green areas respectively represent skeletal muscle, visceral adipose tissue, subcutaneous adipose tissue, and intramuscular adipose tissue.

A single non-enhanced CT cross-sectional image at the level of the third lumbar vertebra (L3) was obtained through the Affiliated Hospital of Qingdao University’s PACS system. The L3 vertebral level has been validated as a reliable reference site for representing whole-body volume and distribution of body composition (20). Manual tissue segmentation was performed using Slicer O Matic software (version 5.0). Tissue compartments were quantified according to predefined Hounsfield unit (HU) thresholds, including skeletal muscle (-29 to +150 HU), subcutaneous fat (-190 to -30 HU), visceral fat (-150 to -50 HU), and intramuscular fat (-190 to -30 HU). The skeletal muscle area encompassed the rectus abdominis, external and internal oblique muscles, and the lumbar paraspinal muscles, and was independently delineated by two radiologists (**Figure 2**). The software automatically calculated both the cross-sectional area (cm^2^) and tissue density (HU). To minimize the impact of body size variations, the area of each compartment was normalized to height squared (m^2^), yielding standardized indices of body composition, including the SMI, SFI, VFI, and IFI.

### 2.4 Data preprocessing and feature selection

Before feature selection, all independent variables were preprocessed. Continuous variables were standardized using Z-score normalization, whereas categorical variables were transformed into dummy variables through one-hot encoding. The preprocessed dataset was then randomly divided into a training cohort and a validation cohort at a 7:3 ratio. Feature selection was performed based on the training cohort. Initially, a univariate analysis was conducted. For continuous variables with P < 0.20, optimal cut-off values were determined using the Youden index derived from the receiver operating characteristic (ROC) curve, after which these variables were dichotomized into high- and low-value groups. Subsequently, variables with P < 0.20 in the univariate Cox regression were further filtered using least absolute shrinkage and selection operator (LASSO)-Cox regression (L1 regularization). The set of features corresponding to the minimum cross-validation error at the optimal λ value was retained. To enhance model parsimony and generalizability, a stepwise Cox regression based on the Akaike Information Criterion (AIC) was finally applied to determine the independent prognostic predictors.

### 2.5 Construction and validation of prognostic models for OS, DFS, and PFS

Based on the final selected variables, a nomogram predicting OS was constructed in the training cohort. Internal validation was performed using 1,000 bootstrap resamples to minimize the risk of overfitting and to assess model robustness. The clinical utility of the model was evaluated in the training set using decision curve analysis (DCA). The utility of DCA is to determine if the prognostic model can help to selectively avoid interventions for low-risk patients, and to identify high-risk ones for extra care. In DCA, three curves are plotted: “Treat all,” representing the scenario where all patients are treated regardless of their risk; “Treat none,” where no patients are treated; and “Model,” which represents the benefit of using the prognostic model to guide treatment decisions. By comparing these curves, we can assess the net benefit of using the model at different thresholds of predicted risk. Model discrimination was assessed by ROC analysis, with the area under the curve (AUC) used as a measure of predictive accuracy. A higher AUC indicates better discriminatory ability of the model in distinguishing between high-risk and low-risk patients. Similarly, nomograms for PFS and DFS were established in the training cohort, and their predictive accuracy was evaluated. All prediction models were subsequently re-evaluated in the validation cohort to confirm their performance and generalizability.

### 2.6 Development and evaluation of the prognostic risk stratification model

The risk score of patients was calculated based on the characteristic coefficient weights of each variable in the nomogram model, and two optimal cut-off values were determined using the “cutoff” package of R software. The patients were then divided into low, medium, and high-risk groups. Subsequently, the Kaplan-Meier survival curve analysis was used to compare the prognostic differences among patients in different risk groups. To further demonstrate the expression distribution of model variables in different risk groups, a corresponding heatmap was drawn. At the same time, a hotspot map was constructed based on the survival status and survival time data of the patients to visually compare the clinical outcomes of each risk group. Finally, ROC curve and DCA curve were drawn to evaluate the predictive performance and clinical applicability of this prognostic risk stratification model.

### 2.7 Statistical analysis

Statistical analysis was conducted using version 4.4.2 of the R software. The Mann-Whitney U test was uniformly used for comparisons between groups of continuous variables, and the results were presented as mean (standard deviation) (SD). Categorical variables were expressed as frequencies (percentages), and comparisons between groups were conducted using the χ² test or Fisher’s exact test. The “dplyr” R package was employed for data preprocessing. The “survival” package was applied to perform Cox proportional hazards regression analysis and fit KM survival curves. The “glmnet” package was utilized for LASSO-Cox regression analysis. Stepwise regression analysis based on AIC was conducted using the “MASS” package. The “timeROC” package was used to compute time-dependent ROC curves. The “cutoff” package was used to identify optimal cut-off values for risk stratification. The “rms” R package was used to generate ROC curves, calculate the AUC, construct a nomogram, and generate the corresponding calibration curve. The “rmda” package was used for DCA. A two-tailed P value < 0.05 indicated statistical significance.

## 3 Results

### 3.1 Baseline characteristics

A total of 403 patients were included in this study, and their baseline characteristics are shown in **Table 1**. The patients were divided into a training set (n=283) and an internal validation set (n=120). Among the patients in the training set, 214 were male (75.6%), and 95 were male in the validation set (79.2%). The average age of patients in the training set was 60.42 ± 9.72 years, and that in the validation set was 58.62 ± 10.04 years. The ypTNM stage distribution in the training cohort was 20 (7.1%) for stage 0, 56 (19.8%) for stage I, 94 (33.2%) for stage II, and 113 (39.9%) for stage III. In the internal validation cohort, 16 (13.3%) patients were classified as stage 0, 33 (27.5%) as stage I, 35 (29.2%) as stage II, and 36 (30.0%) as stage III. Except for ypTNM stage (p = 0.034), no significant differences were detected in other baseline characteristics between the two cohorts (p > 0.05).

**Table 1:** Baseline characteristics of patients in the training and validation sets.

Variables	Training set (N = 283)	Validation set (N = 120)	*P value*
Gender			0.521
Female, n (%)	69 (24.4)	25 (20.8)	
Male, n (%)	214 (75.6)	95 (79.2)	
Age (years), Mean (SD)	60.42 (9.72)	58.62 (10.04)	0.092
Pre-BMI, Mean (SD)	26.17 (2.13)	26.21 (2.71)	0.890
Post-BMI, Mean (SD)	25.27 (2.08)	25.33 (2.44)	0.780
Operation time (minute), Mean (SD)	220.12 (49.28)	219.71 (48.72)	0.938
Blood loss (ml), Mean (SD)	122.86 (82.74)	113.67 (72.92)	0.292
Tumor size (cm), Mean (SD)	4.22 (2.26)	4.07 (2.13)	0.523
CEA (ng/ml), Mean (SD)	30.23 (153.57)	24.43 (94.98)	0.701
CA199 (ng/ml), Mean (SD)	152.15 (622.84)	153.91 (776.20)	0.981
CA125 (ng/ml), Mean (SD)	24.86 (39.13)	20.50 (27.46)	0.268
Hb (g/l), Mean (SD)	121.31 (23.53)	117.45 (24.83)	0.140
Glob (g/l), Mean (SD)	25.87 (3.74)	25.29 (4.06)	0.169
NRS-2002 score			0.440
<3, n (%)	84 (29.7)	41 (34.2)	
≥3, n (%)	199 (70.3)	79 (65.8)	
ASA score			0.679
I/II, n (%)	180 (63.6)	73 (60.8)	
III, n (%)	103 (36.4)	47 (39.2)	
Surgical approach			0.869
Laparoscopy/Robotics, n (%)	181 (64.0)	75 (62.5)	
open surgery, n (%)	102 (36.0)	45 (37.5)	
Surgical scope			0.139
Total resection, n (%)	106 (37.5)	35 (29.2)	
Subtotal resection, n (%)	177 (62.5)	85 (70.8)	
Tumor location			0.657
Upper, n (%)	57 (20.1)	29 (24.2)	
Middle, n (%)	102 (36.0)	42 (35.0)	
Lower, n (%)	124 (43.8)	49 (40.8)	
ypT stage			0.060
T1-T2, n (%)	118 (41.7)	63 (52.5)	
T3-T4, n (%)	165 (58.3)	57 (47.5)	
ypN stage			0.108
N0, n (%)	141 (49.8)	71 (59.2)	
N+, n (%)	142 (50.2)	49 (40.8)	
Alb			0.964
>40.7, n (%)	92 (32.5)	40 (33.3)	
≤40.7, n (%)	191 (67.5)	80 (66.7)	
NLR			0.080
<2.32, n (%)	163 (57.6)	81 (67.5)	
≥2.32, n (%)	120 (42.4)	39 (32.5)	
PLR			0.152
<164.4, n (%)	163 (57.6)	79 (65.8)	
≥164.4, n (%)	120 (42.4)	41 (34.2)	
PNI			0.474
>45.0, n (%)	213 (75.3)	95 (79.2)	
≤45.0, n (%)	70 (24.7)	25 (20.8)	
NRI			0.912
≥97.9, n (%)	262 (92.6)	110 (91.7)	
<97.9, n (%)	21 (7.4)	10 (8.3)	
Complications			0.107
No, n (%)	185 (65.4)	89 (74.2)	
Yes, n (%)	98 (34.6)	31 (25.8)	
SMI			0.742
≥44.0, n (%)	175 (61.8)	77 (64.2)	
<44.0, n (%)	108 (38.2)	43 (35.8)	
IFI			0.845
≥3.6, n (%)	177 (62.5)	77 (64.2)	
<3.6, n (%)	106 (37.5)	43 (35.8)	
SFI			1.000
≥55.82, n (%)	60 (21.2)	25 (20.8)	
<55.82, n (%)	223 (78.8)	95 (79.2)	
VFI			0.333
<43.6, n (%)	172 (60.8)	66 (55.0)	
≥43.6, n (%)	111 (39.2)	54 (45.0)	
CONUT score			0.628
<2, n (%)	127 (44.9)	50 (41.7)	
≥2, n (%)	156 (55.1)	70 (58.3)	
VSR			0.900
≥1.4, n (%)	197 (69.6)	85 (70.8)	
<1.4, n (%)	86 (30.4)	35 (29.2)	
MLR			0.069
<0.198, n (%)	83 (29.3)	47 (39.2)	
≥0.198, n (%)	200 (70.7)	73 (60.8)	
SIRI			0.307
<0.705, n (%)	101 (35.7)	50 (41.7)	
≥0.705, n (%)	182 (64.3)	70 (58.3)	
Comorbidity			0.609
DM, n (%)	29 (10.2)	17 (14.2)	
HP, n (%)	26 (9.2)	13 (10.8)	
No, n (%)	194 (68.6)	78 (65.0)	
other, n (%)	34 (12.0)	12 (10.0)	
TRG			0.166
0, n (%)	20 (7.1)	16 (13.3)	
1, n (%)	52 (18.4)	17 (14.2)	
2, n (%)	122 (43.1)	54 (45.0)	
3, n (%)	89 (31.4)	33 (27.5)	
ypTNM stage			0.034*
0, n (%)	20 (7.1)	16 (13.3)	
1, n (%)	56 (19.8)	33 (27.5)	
2, n (%)	94 (33.2)	35 (29.2)	
3, n (%)	113 (39.9)	36 (30.0)	

*P < 0.05. BMI, body mass index; CEA, carcinoembryonic antigen; Hb, hemoglobin; Glob, globulin; NRS, nutritional risk score; ASA, American Society of Anesthesiologists; Alb, albumin; NLR, neutrophil–lymphocyte ratio; PLR, platelet–lymphocyte ratio; PNI, prognostic nutritional index; NRI, nutritional risk index; SMI, skeletal muscle index; IFI, intramuscular fat index; SFI, subcutaneous fat index; VFI, visceral fat index; CONUT, controlling nutritional status; VSR, visceral-to-subcutaneous fat ratio; MLR, monocyte–lymphocyte ratio; SIRI, systemic inflammation response index; DM, diabetes mellitus; HP, hypertension; TRG, tumor regression grade; ypTNM stage, post-therapeutic pathological TNM stage.

### 3.2 Feature selection and survival analysis

**Figure 3:**
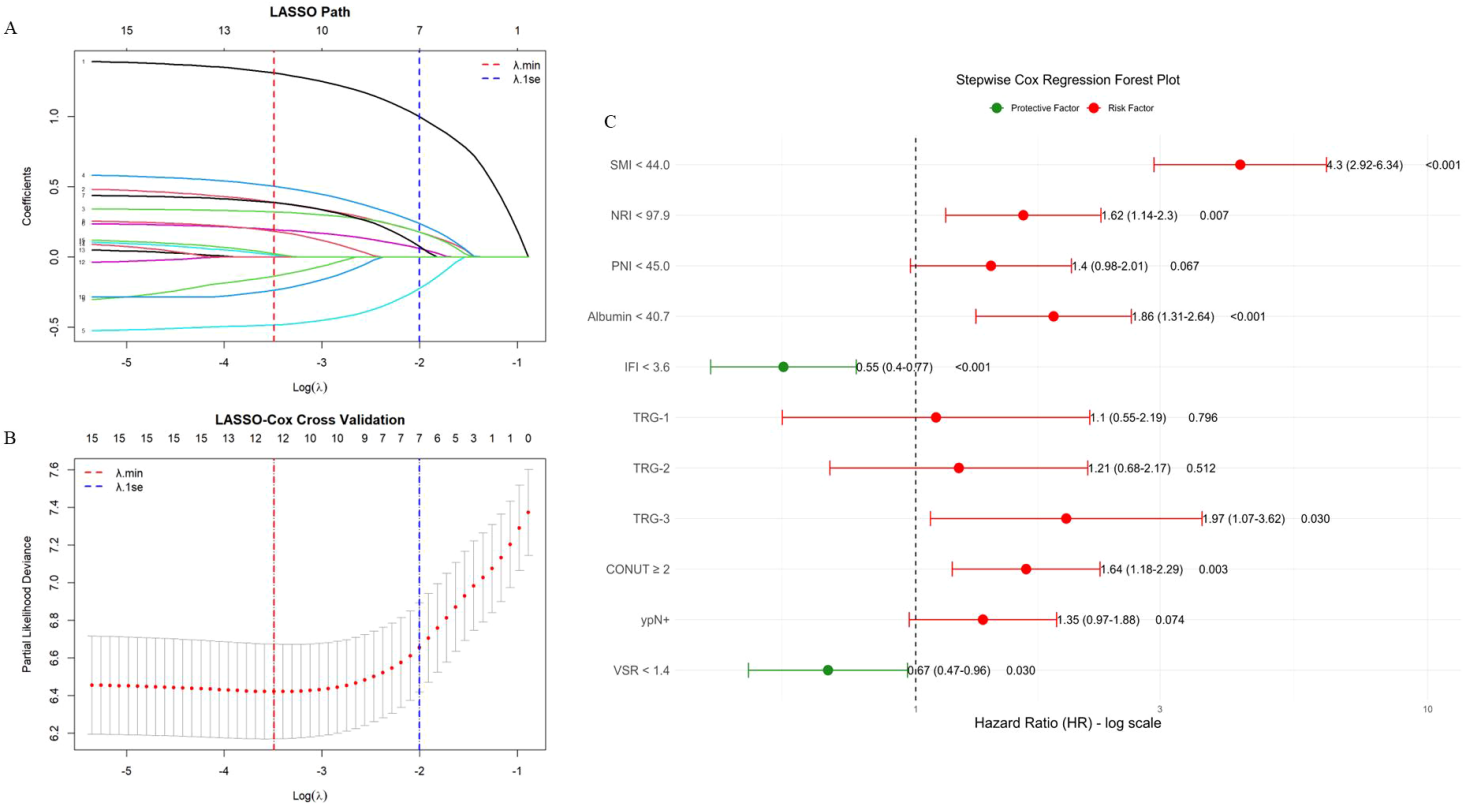
Feature selection graph. **(A)** The LASSO-path graph illustrates the variable selection process, where each curve represents the trajectory of the coefficient of the predictor variable under different regularization intensities (log(λ)). As the regularization parameter λ increases, more coefficients gradually shrink towards zero, representing the effect of regularization on variable selection. **(B)** The ten-fold cross-validation graph is used to determine the optimal regularization intensity. The parameter λ.min corresponds to the value of λ with the minimum cross-validation error, ensuring the most accurate model. Although λ.1se, defined as the value of λ at the minimum cross-validation error plus one standard error, can also be used to select a more conservative model with reduced complexity, we ultimately selected λ.min because it provides the best balance between predictive accuracy and model simplicity. **(C)** The Stepwise Cox Regression Forest graph screens out the final risk factors.

**Figure 4:**
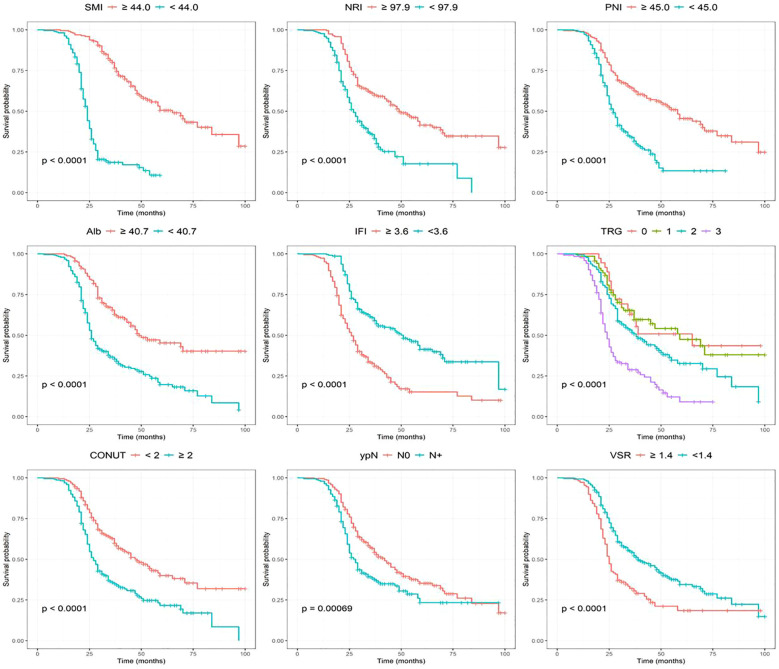
Kaplan-Meier survival analysis was performed based on nine characteristics.

In the training cohort, we first performed a univariate Cox proportional hazards analysis and retained variables with a p < 0.20 (after screening 58 features, 15 features were finally selected for the next stage of analysis). Continuous variables meeting this threshold were subsequently dichotomized using optimal cutoffs determined by the Youden index. To further refine variable selection, we applied LASSO regression to assess feature importance and evaluate the influence of regularization on model sparsity and stability, thereby removing redundant and highly collinear predictors. Based on the LASSO coefficient trajectory (**Figure 3A**) and the ten-fold cross-validation curve (**Figure 3B**), the regularization parameter λ that provided the best trade-off between parsimony and predictive performance was selected. At the optimal penalization level (λ.min = 0.0356), 12 features with non-zero coefficients were identified. Subsequently, stepwise Cox regression using the AIC was applied to determine the nine final model variables, resulting in the inclusion of SMI, NRI, PNI, Alb, IFI, TRG grade, CONUT score, lymph node metastasis status, and visceral-to-subcutaneous fat ratio (VSR) (**Figure 3C**). Kaplan-Meier survival analyses of these finalized predictors demonstrated that low SMI, low NRI, low PNI, low Alb, high IFI, higher TRG grade, elevated CONUT score, lymph node metastasis (ypN+), and high VSR were all significantly associated with worse overall survival (p < 0.01) (**Figure 4**).

### 3.3 Evaluation of OS, PFS and DFS prognostic models

**Figure 5:**
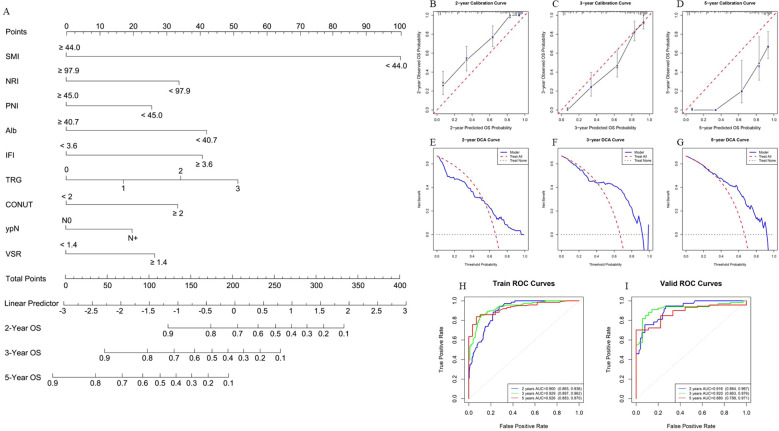
Performance evaluation of the OS prognosis model. **(A)** Nomogram for OS prognosis model. The nomogram visually represents a statistical prognostic model derived from Cox regression for OS outcomes. It plots scales for patient variables (such as SMI, NRI, PNI, Alb, IFI, TRG grade, CONUT score, lymph node metastasis status, and VSR) against points that sum to predict survival probabilities at specific time points (2, 3, and 5 years). This model enables clinicians to predict individual survival probabilities based on patient characteristics, offering a clear visual tool for decision-making. **(B-D)** Calibration curves of the nomogram for 2, 3, and 5 years in the training set. The calibration curves evaluate the accuracy of the survival predictions by comparing predicted survival probabilities with observed outcomes. For example, in **(C)**, the calibration curve for 3-year survival demonstrates good model performance, indicating that the predicted probabilities closely match the observed outcomes. A calibration curve closer to the diagonal line represents a better fit of the model to the data. **(E-G)** Clinical decision curves of the nomogram for 2, 3, and 5 years in the validation set. The decision curves assess the clinical usefulness of the nomogram by considering the net benefit at different thresholds of predicted survival probability. For instance, in **(F, G)**, the decision curve for 3-year and 5-year survival shows that the nomogram provides net benefits across a wide range of threshold probabilities, particularly between 0.5 and 0.9, suggesting that the model is clinically useful in making decisions for a broad set of patients. **(H, I)** ROC curves of the OS prognosis model in the training and validation sets. The ROC curves evaluate the model’s discriminatory ability, with sensitivity plotted against 1-specificity. **(H)** shows the ROC curve for the 2, 3, 5-years OS prognosis model in the training set, where the AUC is 0.900, 0.929, 0.926, indicating excellent discriminatory performance. In the validation set **(I)**, the AUC is 0.916, 0.920, 0.880, demonstrating good generalizability and consistent performance when applied to independent data.

**Figure 6:**
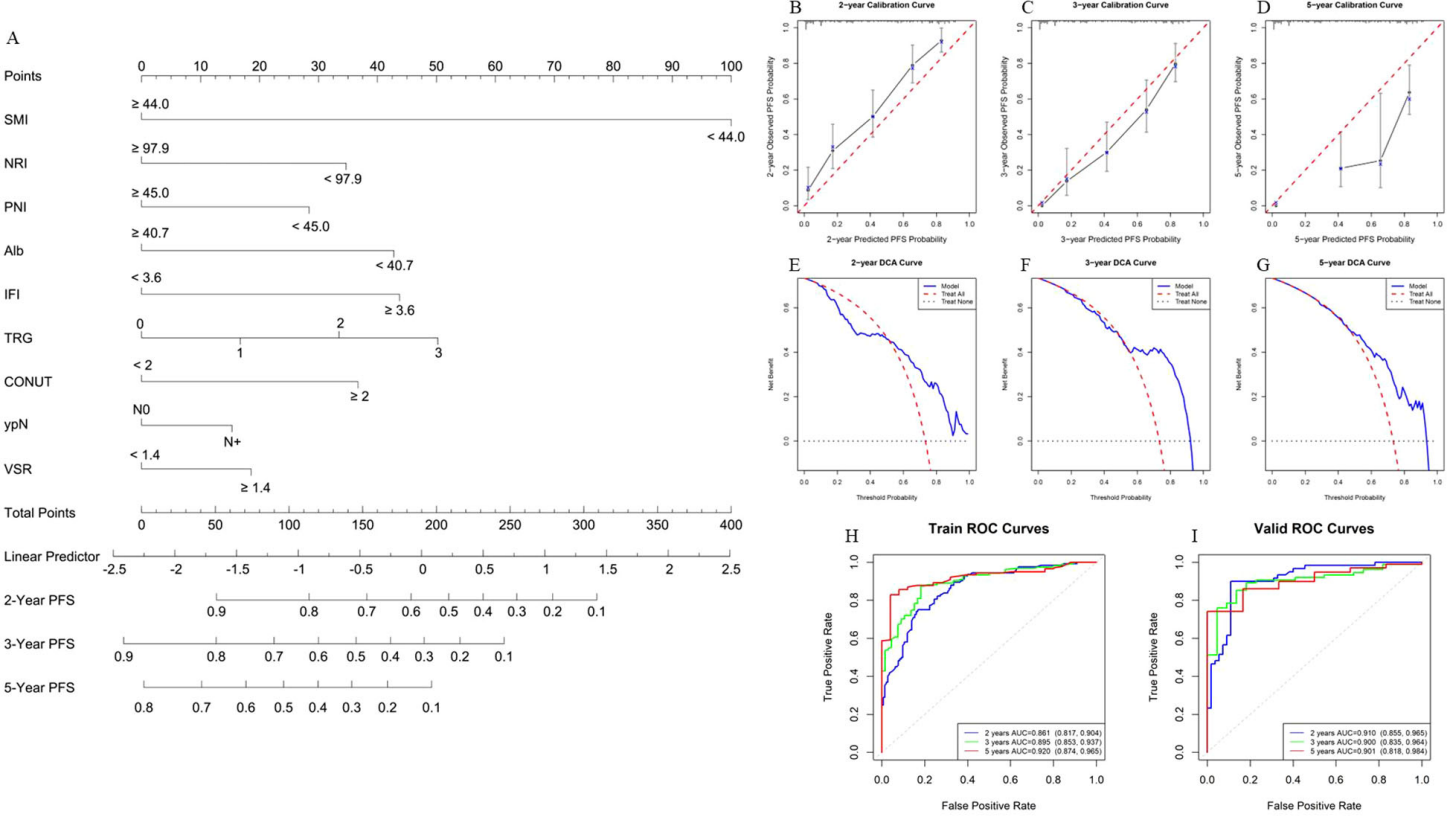
Performance evaluation of the PFS prognosis model. **(A)** Nomogram for PFS prognosis model. The nomogram visually represents a statistical prognostic model derived from Cox regression for PFS outcomes. It plots scales for patient variables against points that sum to predict survival probabilities at specific time points (2, 3, and 5 years). **(B-D)** Calibration curves of the nomogram for 2, 3, and 5 years in the training set. The calibration curves evaluate the accuracy of the survival predictions by comparing predicted survival probabilities with observed outcomes. For example, in **(B, C)**, the calibration curve for 2, 3-years survival demonstrates good model performance, indicating that the predicted probabilities closely match the observed outcomes. A calibration curve closer to the diagonal line represents a better fit of the model to the data. **(E-G)** Clinical decision curves of the nomogram for 2, 3, and 5 years in the validation set. The decision curves assess the clinical usefulness of the nomogram by considering the net benefit at different thresholds of predicted survival probability. For instance, in **(E–G)**, the decision curve for 2, 3, 5-years survival shows that the nomogram provides net benefits across a wide range of threshold probabilities, particularly between 0.7 and 0.9, suggesting that the model is clinically useful in making decisions for a broad set of patients. **(H, I)** ROC curves of the PFS prognosis model in the training and validation sets. The ROC curves evaluate the model’s discriminatory ability, with sensitivity plotted against 1-specificity. **(H)** shows the ROC curve for the 2, 3, 5-years PFS prognosis model in the training set, where the AUC is 0.861, 0.895, 0.920, indicating excellent discriminatory performance. In the validation set **(I)**, the AUC is 0.910, 0.900, 0.901, demonstrating good generalizability and consistent performance when applied to independent data.

**Figure 7:**
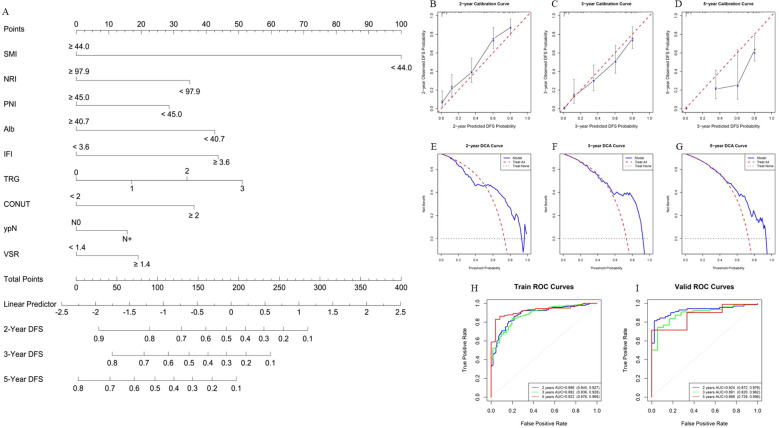
Performance evaluation of the DFS prognosis model. **(A)** Nomogram for DFS prognosis model. The nomogram visually represents a statistical prognostic model derived from Cox regression for DFS outcomes. It plots scales for patient variables against points that sum to predict survival probabilities at specific time points (2, 3, and 5 years). **(B-D)** Calibration curves of the nomogram for 2, 3, and 5 years in the training set. The calibration curves evaluate the accuracy of the survival predictions by comparing predicted survival probabilities with observed outcomes. For example, in **(B, C)**, the calibration curve for 2, 3-years survival demonstrates good model performance, indicating that the predicted probabilities closely match the observed outcomes. A calibration curve closer to the diagonal line represents a better fit of the model to the data. **(E-G)** Clinical decision curves of the nomogram for 2, 3, and 5 years in the validation set. The decision curves assess the clinical usefulness of the nomogram by considering the net benefit at different thresholds of predicted survival probability. For instance, in **(E–G)**, the decision curve for 2, 3, 5-years survival shows that the nomogram provides net benefits across a wide range of threshold probabilities, particularly between 0.7 and 0.9, suggesting that the model is clinically useful in making decisions for a broad set of patients. **(H, I)** ROC curves of the DFS prognosis model in the training and validation sets. The ROC curves evaluate the model’s discriminatory ability, with sensitivity plotted against 1-specificity. **(H)** shows the ROC curve for the 2, 3, 5-years DFS prognosis model in the training set, where the AUC is 0.886, 0.882, 0.922, indicating excellent discriminatory performance. In the validation set **(I)**, the AUC is 0.924, 0.891, 0.868, demonstrating good generalizability and consistent performance when applied to independent data.

Using the finalized feature set, a prognostic model for OS was constructed in the training cohort (**Figure 5A**). Model performance was subsequently assessed using internal validation with 1,000 bootstrap resampling iterations. The bootstrap-corrected concordance index (C-index) reached 0.818, indicating strong discriminative power. Calibration curves demonstrated excellent agreement between the predicted and observed survival probabilities at 2, 3, and 5 years (**Figures 5B–D**). DCA further confirmed that the model yielded a favorable net clinical benefit across a wide range of threshold probabilities (**Figures 5E–G**). In addition, ROC analyses were performed to further evaluate the model^’^s discrimination ability by comparing the corresponding AUC. The ROC curve analysis indicates that the AUC of the training set for 2 years, 3 years and 5 years is 0.900 (95% CI, 0.865 - 0.936), 0.929 (95% CI, 0.897 - 0.962) and 0.926 (95% CI, 0.883 - 0.970), respectively. Also, the corresponding AUC of the validation set is 0.916 (95% CI, 0.864 - 0.967), 0.920 (95% CI, 0.863 - 0.976) and 0.880 (95% CI, 0.788 - 0.971), suggesting that the model has high predictive efficacy and stability (**Figures 5H, I**). Furthermore, we further constructed PFS and DFS prediction models based on the same characteristic variables (**Figures 6**, **7**). The overall results suggest that the constructed prediction models have good discrimination and prediction performance in terms of OS, PFS, and DFS.

### 3.4 Evaluation of the prognostic risk stratification model

**Figure 8:**
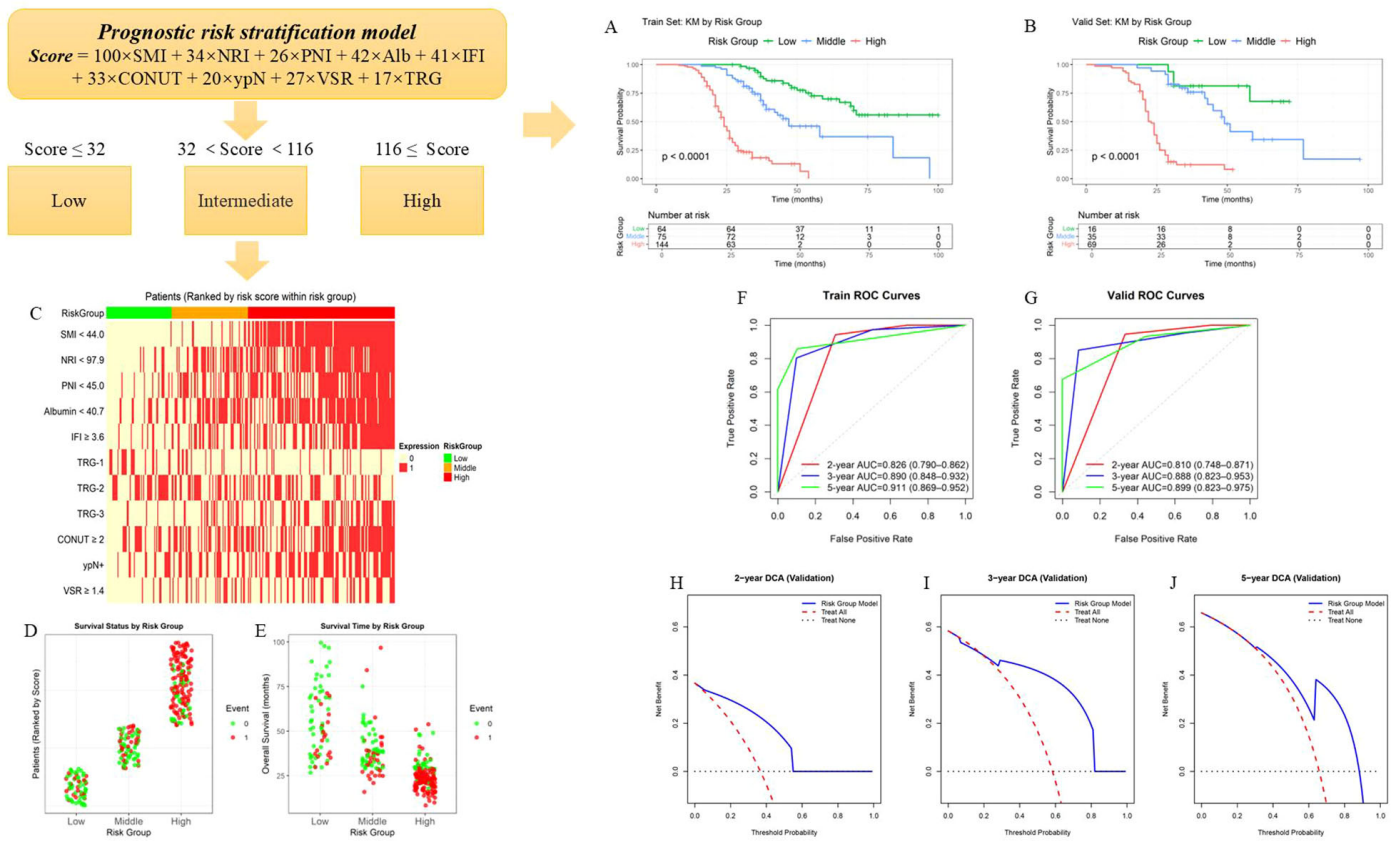
Construction and evaluation of the PRSM. **(A, B)** K-M survival curves of the low, intermediate, and high groups in the training set and validation set. **(C)** Correlation analysis of each predictive indicator with the three risk groups. **(D, E)** Heat maps of the survival status and survival time for each risk group. **(F, G)** ROC curves of the training set and validation set for 2-, 3-, and 5-years survival status. **(H-J)** Clinical decision curves of the PRSM in the validation set for 2, 3, and 5 years.

The feature selection results indicated that SMI, NRI, PNI, Alb, IFI, TRG, CONUT score, lymph node metastasis status, and VSR were independent predictors of prognosis for NAC-LAGC patients. Based on these inflammatory-nutritional indicators, body composition parameters, and clinical pathological features, we constructed a prognostic risk stratification model (PRSM), and then calculated the scoring formula using the weight coefficients of the OS-nomogram to build a three-level risk stratification system to distinguish patients with different levels of poor prognostic risk. The specific formula is as follows: Score = 100 × SMI + 34 × NRI + 26 × Alb + 41 × IFI + 33 × CONUT + 20 × ypN + 27 × VSR + 17 × TRG. (In this formula, SMI ≥ 44.0 as “0”, SMI < 44.0 as “1”;NRI ≥ 97.9 as “0”, NRI < 97.9 as “1”;PNI ≥ 45.0 as “0”, PNI < 45.0 as “1”;Alb ≥ 40.7 as “0”, Alb < 40.7 as “1”;IFI < 3.6 as “0”, IFI ≥ 3.6 as “1”;TRG grade 0 as “0”, TRG grade 1 as “1”,TRG grade 2 as “2”,TRG grade 3 as “3”; CONUT < 2 as “0”, CONUT ≥ 2 as “1”;ypN (N0) as “0”, ypN (N+) as “1”;VSR < 1.4 as “0”, VSR ≥ 1.4 as “1”). The optimal cut-off points (32 and 116) are determined through the “threshold” package in R software, thereby dividing the patients into three groups: low risk (≤32), intermediate risk (32 < score < 116), and high risk (≥116) (**Figure 8**). The Kaplan-Meier survival analysis results showed that there was a significant difference in the survival curves of the three groups of patients in the training set and validation set (p < 0.001) (**Figures 8A, B**). At the same time, a further assessment was conducted on the correlation between the variables included in the model and each risk stratification (**Figure 8C**). Additionally, we further compared the outcomes of patients in different risk groups in terms of survival status and survival time. The results showed that patients in the low-risk group had a relatively ideal overall survival situation, manifested by longer survival time and higher survival rate; in contrast, patients in the high-risk group had significantly shorter survival time and the lowest survival rate; and patients in the intermediate-risk group had survival outcomes between the two extremes (**Figures 8D, E**). To further evaluate the discriminatory ability of PRSM, ROC analysis was conducted in both the training set and the validation set to compare the AUC. The 2-year AUC was 0.826 (95%CI, 0.790 - 0.862), the 3-year AUC was 0.890 (95%CI, 0.848 - 0.932), and the 5-year AUC was 0.911 (95%CI, 0.869 - 0.952) in the training set. In the validation set, the 2-year AUC was 0.810 (95%CI, 0.748 - 0.871), the 3-year AUC was 0.888 (95%CI, 0.823 - 0.953), and the 5-year AUC was 0.899 (95%CI, 0.823 - 0.975) (**Figures 8F, G**). Clinical decision curves of PRSM in the validation set at 2, 3, and 5 years also shows that the model yielded a favorable net clinical benefit across a wide range of threshold probabilities (**Figures 8H–J**). These results further validate the reliability and clinical applicability of the PRSM for prognosis assessment and risk stratification (**Table 2**). By integrating critical markers of nutritional status, such as serum albumin levels, NRI, and skeletal muscle mass, PRSM enhances patient risk stratification, providing valuable insights into postoperative recovery and long-term survival. This model offers improved clinical utility by enabling personalized, targeted nutritional interventions tailored to the patient’s risk profile. In comparison to the traditional nomogram, PRSM facilitates a more individualized approach, optimizing perioperative care and potentially improving outcomes by enabling early identification of patients who require intensive nutritional support or rehabilitation.

**Table 2:** Predictive performance of all models in the training and validation sets.

Model category	2y-AUC (95%CI)	*p value*	3y-AUC (95%CI)	*p value*	5y-AUC (95%CI)	*p value*	C-index
Nomogram model							
Training-OS	0.900(0.865-0.936)	<0.001	0.929(0.897-0.962)	<0.001	0.926(0.883- 0.970)	<0.001	0.818
Training-DFS	0.886(0.845-0.927)	<0.001	0.882(0.836-0.928)	<0.001	0.922(0.876-0.968)	<0.001	0.776
Training-PFS	0.861(0.817-0.904)	<0.001	0.895(0.853-0.937)	<0.001	0.920(0.874-0.965)	<0.001	0.776
Validation-OS	0.916(0.864-0.967)	<0.001	0.920(0.863-0.976)	<0.001	0.880(0.788-0.971)	<0.001	0.826
Validation-DFS	0.924(0.872-0.976)	<0.001	0.891(0.820-0.962)	<0.001	0.868(0.728-0.998)	<0.001	0.788
Validation-PFS	0.910(0.855-0.965)	<0.001	0.900(0.835-0.964)	<0.001	0.901(0.818-0.984)	<0.001	0.787
PRSM							
Training	0.826(0.790-0.862)	<0.001	0.890(0.848-0.932)	<0.001	0.911(0.869-0.952)	<0.001	0.836
Validation	0.810(0.748-0.871)	<0.001	0.888(0.823-0.953)	<0.001	0.899(0.823-0.975)	<0.001	0.829

## 4 Discussion

This study analyzed the multi-dimensional clinical indicators mainly related to nutrition of NAC-LAGC patients before radical gastrectomy, aiming to explore the risk factors associated with long-term poor survival outcomes and to construct a prognostic prediction model. The results showed that low SMI, low NRI, low PNI, low Alb, high IFI, poor TRG classification, high CONUT score, ypN+, and high VSR were all closely related to the poor prognosis of NAC-LAGC patients. We further integrated these multi-dimensional predictive factors related to nutrition to construct a long-term survival prognosis model for NAC-LAGC patients, and further constructed a simple and interpretable risk stratification model for clinical application, providing a basis for perioperative nutritional intervention and individualized treatment strategies.

GC is frequently accompanied by impaired digestive function, which compromises food digestion and nutrient absorption and predisposes patients to chronic malnutrition and unfavorable clinical outcomes (21, 22). Although the tumor-suppressing efficacy of NAC has been repeatedly validated and shown to improve long-term survival, as evidenced by a meta-analysis reporting an OS rate of 41.6%-74.2% in patients receiving neoadjuvant therapy compared with 30.9%-74.0% in untreated patients (23), the potential nutritional burden induced by NAC remains a major clinical concern. Cytotoxic agents used in NAC commonly induce gastrointestinal toxicities, including nausea, vomiting, anorexia, and delayed gastric emptying, which directly reduce dietary intake and accelerate the decline in nutritional reserves (24). Additionally, patients with LAGC often experience a hypermetabolic and catabolic state during NAC, leading to disproportionate depletion of muscle and adipose tissues, resulting in progressive alterations in body composition. This deterioration of nutritional status further impairs immune function and reduces host antitumor capacity, thereby increasing susceptibility to tumor progression and cancer-related mortality. Given this bidirectional interaction between nutritional deterioration and oncologic outcomes, timely and accurate nutritional assessment during the perioperative period is of particular clinical importance. Recent studies have emphasized the prognostic value of composite nutritional indices that integrate immune-inflammatory and metabolic markers, such as the PNI, NRI, and CONUT score. For example, Ding et al. identified the SII-PNI score as an independent predictor of progression-free survival (P = 0.003), while Li et al. reported that both elevated NRI (C-index = 0.737; 95% CI, 0.701-0.774) and CONUT scores (C-index = 0.635; 95% CI, 0.597-0.673) were associated with poorer survival outcomes (7, 25). Collectively, these findings highlight the necessity of incorporating perioperative nutritional evaluation into clinical decision-making to optimize the prognostic management of NAC-LAGC patients.

Traditional nutritional assessment tools, such as body weight, BMI, and NRS-2002 scores, are frequently influenced by subjective evaluation, hydration status, and short-term physiological fluctuations, which may underestimate or delay the detection of clinically significant nutritional decline. In contrast, imaging-based assessment of body composition provides an objective, anatomically grounded, and quantifiable approach for nutritional evaluation. By accurately distinguishing skeletal muscle, subcutaneous fat, and visceral adiposity, body composition analysis can reveal pathological patterns such as abnormal fat distribution and occult sarcopenia, which are often undetected by conventional screening tools (26, 27). Moreover, body composition metrics reflect long-term nutritional reserve rather than transient inflammatory or stress-related changes, thereby offering greater prognostic stability compared with serum-based inflammatory-nutritional indices. Increasing evidence supports the prognostic value of body composition in oncology. For instance, Gezer et al. demonstrated that composite body composition indices could effectively predict 3-year recurrence risk in lung cancer patients (AUC = 0.780, 95% CI: 0.750-0.830) (28). Similarly, Lazar et al. emphasized the clinical necessity of developing a practical and cost-efficient prediction tool that integrates body composition with additional biomarkers to enhance cancer mortality risk stratification (29). These findings suggest that body composition-based assessment offers a promising supplemental strategy for prognostic evaluation in cancer patients. Accordingly, exploring its predictive relevance in NAC-LAGC patients may provide novel insights into nutritional risk classification and individualized clinical management.

The feature selection results of this study indicated that high CONUT score, low albumin level, low NRI, low PNI, poor TRG rating, lymph node metastasis (ypN+), low SMI, high VSR, and high IFI were adverse prognostic factors for NAC-LAGC patients. Among these variables, the CONUT score is a widely used nutritional screening tool derived from serum albumin, total cholesterol, and total lymphocyte count (18). The lymphocyte count reflects the intensity of the host immune response, while albumin level serves as an indicator of protein reserves and overall nutritional status. Reduced cholesterol may compromise antitumor immunity, thereby contributing to unfavorable oncologic outcomes (30). Hypoalbuminemia often results from malnutrition, hypercatabolism, or systemic inflammation, and further suppresses immune function, which facilitates tumor proliferation, local invasion, and infectious complications, ultimately worsening patient prognosis (31, 32). The NRI integrates serum albumin and body weight to reflect both nutritional and inflammatory status. A decreased NRI is associated with impaired liver function reserve and compromised immunity, leading to suboptimal treatment outcomes, and is therefore recognized as an important prognostic indicator in cancer patients (33). Likewise, a low PNI indicates protein deficiency and immunosuppression, which reduces the host’s capacity to eliminate residual tumor cells, increasing the likelihood of recurrence/metastasis and shortening survival (34). TRG reflects the histopathological response to systemic therapy. A high TRG (indicating poor response) suggests chemoresistance or adverse biological tumor behavior associated with invasion and metastasis, leaving residual tumor burden that predisposes to local or distant recurrence (35). Similarly, ypN+ status indicates persistent nodal metastasis after neoadjuvant chemotherapy, suggesting limited chemosensitivity and a greater propensity for systemic dissemination, which markedly increases the risk of postoperative recurrence and distant metastasis (36).

Recent evidence highlights that alterations in body composition, particularly low SMI, high VSR, and high IFI, are important determinants of cancer prognosis (37). Yuhei Hamaguchi et al. demonstrated that patients presenting with low SMI and high VSR experienced significantly shorter postoperative survival, underscoring the prognostic relevance of both muscle depletion and abnormal fat distribution (38). Low SMI reflects quantitative skeletal muscle loss, leading to sarcopenia, which has been widely recognized as an independent predictor of adverse outcomes in malignancies (39). Sarcopenia impairs physical reserve, reduces tolerance to surgical and systemic treatments, and increases susceptibility to treatment-related toxicity (40–42). However, muscle quantity alone does not fully explain the prognostic value of skeletal muscle abnormalities. High IFI suggest qualitative deterioration due to intramuscular fat infiltration. Such fat infiltration disrupts mitochondrial function and energy metabolism, triggers insulin resistance and oxidative stress, and ultimately compromises muscle performance and treatment tolerance (43). Furthermore, alterations in muscle composition may modify the pharmacokinetics of antitumor agents, increasing chemotherapy toxicity and diminishing efficacy (44). In parallel, a high VSR reflects disproportionate visceral fat accumulation, which promotes a pro-tumor metabolic and immune microenvironment. Visceral adiposity induces systemic inflammation and upregulates programmed death receptor-1 (PD-1) expression, leading to T-cell exhaustion and impaired anticancer immunity, thereby facilitating tumor progression (45). Taken together, low SMI, high VSR, and high IFI each represent distinct but interrelated aspects of body composition abnormalities, collectively contributing to poor prognosis through impaired metabolic regulation, reduced treatment tolerance, and disrupted immune function.

Current prognostic studies have predominantly relied on conventional nutritional assessment indices. Although these indicators partially reflect patients’ physiological status, their independent use often results in insufficient accuracy and limited clinical applicability, failing to meet the growing demand for individualized and comprehensive perioperative management (46–48). In response to these limitations, an increasing number of investigations have shifted toward multivariate models integrating nutritional, inflammatory, surgical, and tumor-related features to enhance prognostic performance. In this context, we constructed a multidimensional prognostic model that uniquely incorporates body composition-related nutritional indicators combined with other predictive clinical factors, thereby reflecting both the metabolic reserve and systemic status of patients. Notably, our model demonstrated superior discrimination in predicting long-term survival, with AUC values consistently exceeding 0.80 across 2-year, 3-year and 5-year outcomes in both the training and validation cohorts (Training set: 0.826, 0.890, 0.911; Validation set: 0.810, 0.888, 0.899). This robustness across multiple time points highlights its stability and potential generalizability in clinical practice. Previous studies have also attempted to integrate body composition into prognostic models. For example, Tao et al. developed a nomogram using ΔBMD, ΔVFA (Visceral Fat Area), ΔCTFF (Computed Tomography Fat Fraction), and preoperative PNI with a C-index of 0.743 (49). Zhong et al. incorporated SMD-related measures with PNI and achieved a C-index of 0.714. However, these models mainly emphasized single functional domains of body composition (e.g., muscle attenuation or adipose tissue change) or focused only on selective nutritional metrics, thus lacking a panoramic reflection of patient nutritional heterogeneity. In contrast, our model simultaneously captures structural, metabolic, and functional dimensions of nutritional status, providing a more comprehensive assessment that better mirrors tumor burden, metabolic consumption, and treatment-induced physiological variations. Collectively, the present findings underscore that strategically integrating detailed body composition metrics with clinical nutritional indicators can substantially improve risk stratification and prognostic accuracy. This multidimensional approach has important implications for guiding individualized perioperative nutritional support and optimizing clinical decision-making in GC.

While the calibration curves for 5-year survival show a greater deviation between predicted and observed survival compared to the 2-year or 3-year survival, it is important to note that this discrepancy does not necessarily indicate a substantial limitation of the model. The predicted survival at 5 years tends to underestimate observed survival, which may be partly due to the inherent uncertainty and the fact that survival predictions over longer horizons, such as 5 years, are generally more difficult and prone to greater variability. However, when considering the ROC curves and AUC values in the validation cohort, the model still demonstrates acceptable discriminatory performance at 5 years, with AUC values remaining strong and consistent with the model’s overall ability to distinguish between high and low-risk patients. This suggests that while the calibration curve for 5-year survival exhibits some divergence, the model’s predictive accuracy at this time point remains clinically relevant. We acknowledge that the model’s performance is more precise at shorter time frames, such as 2 years, which is closer to the time of potential nutritional interventions. Therefore, while using 2-year survival data could increase precision, the observed 5-year survival performance does not significantly compromise the model’s clinical utility. The overall model remains useful for both short-term and long-term predictions.

Preoperative nutritional intervention optimizes patients’ metabolic and physiological status, thereby improving postoperative recovery and conferring potential long-term survival benefits. Based on the risk stratification established by our model, individualized perioperative nutritional strategies can be more precisely implemented. For the medium-risk group (32 < Score < 116), dynamic monitoring of serum albumin levels is recommended, combined with a high-protein, high-calorie diet to ensure sufficient energy and protein intake. In addition, resistance training and early rehabilitation programs should be incorporated to preserve skeletal muscle mass and facilitate postoperative functional recovery. For the high-risk group (Score ≥ 116), intensified nutritional support should be initiated 7–10 days before surgery. Oral nutritional supplements (ONS) are recommended as the first-line intervention, while enteral nutrition (EN) or short-term parenteral nutrition (PN) should be considered for patients with inadequate oral intake or impaired gastrointestinal function (50). A randomized controlled trial demonstrated that short-term intensive nutrition before surgery significantly reduces postoperative complications in gastric cancer patients (51). Notably, for patients characterized by elevated IFI and VSR, anti-inflammatory nutritional strategies may be particularly beneficial, such as supplementation with omega-3 fatty acids, glutamine, and antioxidant nutrients, which may attenuate inflammation, modulate the microenvironment, and enhance immune function. For the low-risk group (Score ≤ 32), perioperative nutritional surveillance should be emphasized, with timely reassessment to guide further nutritional optimization if clinically required. Taken together, the multi-index risk prediction model developed in this study provides a practical framework for precision nutritional management. By facilitating targeted and multidisciplinary preoperative nutritional planning, this strategy has the potential to optimize patients’ systemic condition and ultimately translate into improved long-term survival.

Although this study has taken adequate considerations in its design and methods, there are still several limitations. Firstly, as this study is a single-center retrospective analysis, selection bias is difficult to completely eliminate. Therefore, it needs to be verified through prospective and multi-center studies. Secondly, patients have differences in their family dietary habits and lifestyles, and the postoperative recovery speeds vary. Therefore, it is difficult to clearly determine the specific impact of the decline in nutritional status after discharge caused by individual factors on prognosis. The exclusion of patients with severe infections or metabolic disorders prior to surgery may introduce selection bias, as these conditions are often associated with nutritional issues and inflammation related to tumor progression. As a result, the generalizability of our findings may be limited for patients with such comorbidities. Future research should include these patients to evaluate the model’s performance in a more diverse and representative population. Also, future research should strengthen long-term follow-up of patients, expand the sample size, and combine pathological section analysis and multimodal imaging assessment to more comprehensively and dynamically reflect the nutritional status and body composition changes of patients. In addition, molecular biomarkers and immunological indicators can be included in the assessment to explore the potential impact of nutritional intervention on the tumor microenvironment and prognosis, providing a more solid evidence-based foundation for the formulation of individualized treatment strategies.

## Data Availability

The raw data supporting the conclusions of this article will be made available by the authors, without undue reservation.
